# On Pseudoplasmata

**Published:** 1858-07

**Authors:** Alonzo L. Cartier

**Affiliations:** Solcure, Switzerland.


### AMERICAN JOURNAL

OF

### DENTAL SCIENCE.

Vol. VIII. NEW SERIES—JULY, 1858. No. 3.

### ORIGINAL COMMUNICATIONS.

### ARTICLE I.

*On Pseudoplasmata.* By Alonzo L. Cartier, M. D., D. D. S., of Solcure, Switzerland.

In perusing this paper the reader will find himself directed to a subject, the intimate relations of which to dental practice it is scarcely necessary to point out. As it, however, is connected with affections, a thorough knowledge of which is almost entirely restricted to those who have opportunities of acquiring it by a liberal clinical supply and careful observation, it would be well before entering upon its consideration, to advance some preliminary remarks.

The information the writer received about statements laid down hereafter, must be carried back to the termination of his medical studies at Heidelberg, Prague, and lastly at the University of Vienna, where, upon more than three years attendance at the general hospital, and for what is confined in particular to the surgical department, the most active interest was taken by him in observing the above named affections.

Nor was it but for the daily and permanent presence and participation at the researches made, as well as at the operations performed, the assistance in the no less troublesome microscopic examination and chemical analyses sought after, was always kindly offered, and he availed himself of the later ones in the pathologico-chemical laboratory of Dr. Heller. In this way it was for some part, at least during the course of unremitting practice, that facts were previously gathered, and subsequently published by the distinguished surgeon and highly esteemed Professor Schuh, to whom, and to the illustrious principals of the dental institution in this country, I thus tender some tribute of lasting indebtedness.

The very first steps taken in our specialty, lead to the practical conviction of the intrinsic importance of a thorough knowledge of general medicine. It was during an intercourse of several months with the American dentists at Paris that the fact just referred to, presented itself most forcibly in the case of a patient in care of Professor Maisonneuve, at the hôpital de Pitié, afflicted with a formidable cancerous tumor, causing the entire ablation of the inferior maxilla, replaced with an artificial substitute, thus restoring the functions of mastication and speech. The extraordinary skill displayed in this case, both by surgery and dentistry, in the hands of Messrs. Fowler and Préterre, gave rise to numerous learned debates in the Academie Impériale de Médecine, and gained for the above named gentlemen much flattering distinction.

Of this case, as well as of a great many others, the dental journals have not failed to take due notice.

The pathological conditions of the human organism frequently exhibits new formations, presenting a certain high degree of organization. The source from whence they originate is generally two-fold: either they arise as a consequence of local inflammation, the product of which transforms itself into a tissue like the scarred tissue in soft parts, the callous in fractures of the bones, &c.; or where there was previously no inflammation at all, then their origin lies merely in an anomaly of nutrition, brought on by changes in the formative principle. Now, as many of the last named have the property to grow and develop themselves into tumors, they may disfigure only, or bring about functional disturbances, or even endanger life. To those the name of "After rises,"—"New formations,"—"Pseudoplasmata," is applied, because of their being organized new formations, of the origin and growth of which we perceive peculiar modifications of the forming and nourishing principle.

Yet, the distinction as above, however viewed, is in no way fully sufficient, for there are also some few exceptions where pseudoplasmata originally develop themselves under inflammatory symptoms. It furthermore does not afford precise limits between pseudoplasmata and hypertrophies; in the last named of which the quantitative variations in the act of nutrition are predominant. We really may, sometimes, come across pseudoplasmata, their origin evincing great affinity to hypertrophies, when it is only by their further development that we become aware of their true nature, thus exhibiting also variations about the quality in composition. It is, therefore, quite evident, that we are not able to separate pseudoplasmata strictly, from related affections; just as this is the case in regard to other classes of diseases. Nature in her productions, whether sound or diseased ones, infinitely inventive, never seeks for sharp distinctions; on the contrary, shows everywhere transitions by degrees, in such a way that the uselessness of any force put upon her, by any artificial system, must be apparent.

Out of these difficulties, though in a practical point of view of little value, it remains to state in this place, that the designation of "tumors," commonly used in regard to affections above named, is altogether inadequate, for this expression is extremely ambiguous, indicating at one time, a symptom of disease; at another, the disease itself. But in examining even a symptom of disease, it becomes necessary, if we would avoid a contradiction of the prevailing practice in the language, to assign to them a fixed understanding. Thus deriving the word "tumor" from its root: "tumefy;" with but the sense and meaning of distended normal tissue, being apt to diminish again in circumference, as this happens, for instance, after inflammation, the term would not be strictly applicable. If, on the other hand, the said expression might be objected to, as to its pointing out an increase in volume, effected by a collection of serum or blood in cellular tissue, or in a cavity already existing, for the same reason as it would be done in the case where some parts perhaps are lifted up by organs laying faultily beneath, then we are mistaken, because the expression tumor could never be used in advance to the diagnosis, it being impossible, at the time we appreciate the symptoms of disease, to know at once whether or not the increase in volume has been carried out by the elevation of tissue capable of becoming increased or diminished. After such an idea, tumor might rather signify a form of affection, than any of its symptoms. Furthermore, it contradicts itself with the daily use of the expression, for we are told of a tumor in the inguinal region, where the existence, either of a hernia or bubo, has to be determined; of a tumor, when the hip-bone has been luxated in a backward direction; of a tumor, after crushing and extravasation of blood; of peeling out a tumor, its tissue, &c.

As above stated, it would be useless to attempt to do away with this term, we shall, therefore, hereafter use it in its double sense. As a symptom of disease, it must be understood for every increase in volume in any part of the body, however the tumor may have arisen; thus the expression only refers to a change of situation, the borders and contours of superficial or deep-seated parts. In another sense, tumor points out the mass itself, which effects change of form and volume. Now, it is perceived, that every pseudoplasma may, indeed, be called a tumor, but not every tumor a pseudoplasma, and that their usual discussion under the head of tumors, offers many points of objection.

There are different ways in which new formations originate, in particular, as to their germ layer. The most frequent kind of development goes on in the interspaces of elementary parts of normal tissue, either at one or many points together. While the pseudoplasma grows, the bordering normal parts are forced away, and by pressure reverted and absorbed. If the new mass is very voluminous, the original tissue may entirely disappear, and the former takes the place of the latter. It is in this way, for instance, that epulis sometimes occupies the superior maxilla. Very often they arise also on the surface of the skin, and the serous as well as mucous membrane, without taking root from the beginning into their tissue. Still another kind of development takes its origin even in the elementary parts of normal tissue, by real transformation of some physiological texture. There can be no doubt about this fact, as may be judged by analogies; many physiological tissues transform themselves into others, such as muscle fibrils into cellular tissue and fat, cartilages into bones, etc. In regard to pseudoplasmas, the same was observed on the colloyd cysts of the thymous gland, on the carcinoma of the liver, and the globules of swollen lymphatic glands in the neighborhood of carcinomas. In all these cases the process is carried out by endosmose, like the transformation of a pseudoplasma into another, which, although of rarer occurrence, has also to be placed here. Warts, fibroids of benign nature, even a lymphatic crop, as was observed, may, under the influence of existing individual cachexy of this kind, pass over into carcinomas.

Organized products of inflammation are also very apt to produce pseudoplasmas. A cancer, for instance, has been extirpated, the wound seems to heal well, and the granulation has quite a satisfactory appearance, in respect to color, form and resistance. In advance, however, to final cicatrization, the fleshy excrescences become paler, firmer, yellowish, because the cells of the new arising adipose tissue, instead of being formed into fibrils, receive the deposited germ fluid of the cancer. As long as the forming matter of the blood is modified by the inflammatory action, the granulation will be a normal one; as soon, however, as this process comes to an end, the remaining disease of the blood and germ fluid are the principal factors in the act of organization, and explain why the forming excrescences degenerate into pseudoplasma. Under these circumstances, just the same may happen in scarred tissue, as it shows the origin of pseudoplasmas on parts of the body which, after inflammation, have remained indurated.

In the last place, we have to notice their origin out of the blood into the blood-vessels. This fact refers only to the most deleterious new formations, while in connection with it there exists a disease of the blood, and whenever the anatomical elements of the globules and cells have forced their way into the blood, effecting coagulation. The process goes on, either by means of lympathic vessels, or by imbibition of the fluid blastema, or by absorption, through vessels which, by laceration having accidentally or in some other way taken place, are apt to receive the globules and cells, or by growing exuberantly on the part of the pseudoplasma into a vein. This kind of development may, for the most part, be found in the lungs, liver and spleen, either of which depends upon the original place of the cancer, as the blood circulation of the veins carries the diseased matters to the lungs, that of the arteries easier to the spleen, and that of the vena porta to the liver.

By the foregoing remarks it may have been anticipated that the blastema of new formations, also called cystoblastema, takes its origin out of the blood, wherefrom all matters liable of organizing themselves are secreted in the adjacent parts, either by way of transudation or exudation, or by escaping through lacerated vessels, or by coagulation inside of the blood-vessel. Their chemical composition, in all these cases, has not been cleared up, yet there can be no doubt that fibrin and albumen are the most important factors in the act of organization, however they may become modified or otherwise chemically changed.

The above named different ways of development account for the many varieties of tissue arising from the blastema, but these are, moreover, influenced by the blastema itself, and by the force and peculiar kind of effects produced by adjoining organs on the blastema. These effects from the neighboring parts, where the blastema has been deposited, are by no means always the same. They modify, just in proportion to the more or less firm connection of such parts with the organic surrounding tissues, depend upon the energy and action of such organs and their tissue, their functions, the influence of the nerves and their relations to external effects. Now, as either the one or the other of both factors of organization prevails at the particular time, it explains why different blastemas in the same organ result sometimes into a fibroid, fibrinous cancer, or fungous carcinoma. On the other hand, it further elucidates the fact, that the very same blastema, in different or the same organs, becomes transformed into a cartilaginous tumor, a mass of adipose tissue or a carcinoma, depending in the same degree upon different vitality and influence of nerves. Thus we may appreciate too, how it is that the existence of the same kind of pseudoplasma sometimes is followed by local, sometimes by general consequences, that very often it originates simultaneously in different organs, and makes its appearance in the uterus, for instance, for the most part under the form of fibrinous tumor, in the bones as enchondroma, and on the tongue as epithelial carcinoma.

Equally different is the act by which the blastemas organize themselves into tissues, as it goes on, either in a fluid state or by coagulation. Fibrin, as well as albumen, may indeed remain in a fluid state or coagulate, and both depends upon the existence, quantity or deficiency of such commixtures of salt and fat as may prevent coagulation, in some points perhaps also upon peculiar chemical modification of these forming matters.

Whenever blastema, its condition being a fluid one, becomes organized into a pseudoplasma, then this process, at least in a great many cases, is carried out by certain physiological laws, which have been pointed out by Schwann and Schleiden: i. e. the development of elementary granuli, nuclei and cells in the germ fluid. The first one by growing becomes metamorphosed into a nucleus, wherefrom a nucleolus develops itself by division, and is the parting point for cells, by means of its being differentiated. Out of these elements another cellular form frequently makes its appearance, called the granulated cell and the stalk, which need some more consideration, inasmuch as their further development variously changes.

What we mean by granuli or globuli are plain, roundish bubbles or vesicles, shaped like the blood-globules, their contents clear or mixed with molecules. For the most part they arise inside of one to three granular corpuscles, and are called naked in case of absence of any cellular integument. If the said membrane exists, however, the designation of cell-granuli is applied, and, in this condition, they may either remain, or in connection with other elements compose a pseudoplasma; or their shape becomes changed, and they are transformed into fibrils. Globuli, which string themselves after their length, give rise to the so-called varicose fibre, and after the knot has disappeared, to the granular one, of which the elastic fibre, with sharp boundaries and a tendency to a twisted course, but without known origin, must be separated. By intususception of the welting blastema the globules are apt to increase in volume, and then appear like blisters, wherefrom the cyst is formed in after-time. There are also globuli of the same character, which by growing touch almost every point of the integument; they are called blistered, and may sometimes fully unite with the cellular integument, thus forming the wall of cysts.

The cell is of vesicle-like formation and different size, consisting of an integument, wherein the content, with one or more granuli, is enclosed. Division was made between their exogen and endogen development, because in the former the cell arises out of the globules in the manner just described, while, in the other way, it develops itself from a globule bound to a cell already existing. Still another kind strikes the observer in the formation of granular cells, mostly found in exudations; elementary globuli gather together and form the integument aforesaid.

The stalk, as above referred to, is another element possessing all the properties for producing, either alone or in a secondary degree, new formations. It consists of an integument without perceptible structure, in the beginning clear and transparent, but lost afterwards because of the presence of globuli and cells, and originates on serous and slimy coats, the inside of cysts and many other places. Its length sometimes has been approximated to $\frac{1}{250}$ line, while in shape it represents a rag or prolonged sac, or a shallowy strip of tissue, or a structure of dentritic vegetation. Many formations, such as adipose tissue, fat cells, layers of vesicles, parenchymatous mass and vessels grow out of this element, and are found in the different kinds of pseudoplasmata.

It is very doubtful whether torpid blastemas alone form a new formation; in the majority of cases, at least, they always appear in connection with fluid ones. Their shape is very different, sometimes not unlike a mass similar to jelly, in which, by splitting to fibrils all around the vesicles, the granular texture originates, or like a dense coat, as observed in the walls of many parenchymatous cysts, or a solid ragged mass interrupted by defects, as in the just named cysts, when occurring near the angle of the inferior maxilla. At whatever time these forms of coagulation organize themselves, it is always done by splitting to fibrils, and reminds us very much of the coagulation of blood and secretion of fibrous matter in the large vessels and cavities of the heart.

Far from being disorderly cast together, as might be presumed, the forming elements of pseudoplasmas on the contrary, give evidence of their different or even remarkably constant proportions to each other, as it regards their reciprocal layer, and for the reason, that the very tissue is based upon the arrangement of the forming elements, its true comprehension must be regarded as the foremost purpose of microscopic research; this we may frequently find out with ease, in many cases however with much difficulty, in still others not at all.

Quite a number of pseudoplasmas consists of one and the same kind of forming elements, like, for instance, the first form of epithelial carcinoma; others, such as several of the fungous cancers, only present granuli beside the germ fluid, and some are built up in fibrils. In most of them, the various forming elements are found together, but we frequently observe, that these dissimilar last named ones are, in some way or other, separated, and their layers almost regularly pushed one into the other. As the result of these observations, any such part possessing a denser consistency in comparison with the other, as if rendering him support, is called stroma, the other content, either of which may prevail. The former appears meshy, areolar, shaggy, or papillous, while the contents are found to be variously colored fluid, as in most pseudoplasmas, blood in fungous hæmatodes, or a torpid mass in the gallertic carcinoma, etc.

Another texture, of the same frequent occurrence, both in benign and malign pseudoplasmatas, which calls for our attention, is named granular or alveolar, and takes its origin where cysts or vesicles without dictinct structure are imbedded in parts full of elements constituting adipose tissue. Such elements are the nuclei and cells. The former elongate themselves, while the others become changed into delicate, clear and roundish fibrils, enclosing an oblong nucleus; both join together closely to the circumference of the vesicle, and during the growth they become transformed into real adipose tissue. This, then, perfectly or imperfectly developed, surrounds the vesicle or cyst, in the shape of a capsule, and by growing together with the wall of the vesicle forms the proper wall of the cyst, while, on the other hand, the inter-alveolar adipose tissue represents curved bundles of fibrils and communicating spaces. Vesicles of this kind, with their capsules, regularly lay very near each other, are now and then filled with nuclei and cells, and are frequently noticed to be enclosed by a still greater capsule. Although this structure cannot be pointed out in very delicate formations, unless with much difficulty, yet there are many cases in which the fact is proved at once, that granular texture is formed, called also alveolar, and, in particular, it refers to the gelatinous tumor and carcinoma.

Besides what has already been said about the tissue of pseudoplasmata, it is remarkably different, according to the period of development; the transition of one pseudoplasma into another, and the various points of the tumor. Thus we may become aware that the development has not everywhere progressed in the same degree, that there are, perhaps, only some points where a change has taken place, or the tissue is partly diseased and destroyed by entirely rotten tubercle like mass, etc., or there exists, in fact, a primordial composition of tissues.

It is after their tissue, borders and relation to the blood, that pseudoplasmata have been divided into homöoplasies and heteroplasies, into formations strictly separated from normal tissue, or which present themselves under the form of infiltration, and lastly into such of benign or malign character.

Of the first named, the former are nothing else than repetitions and imitations of normal tissue, with which the latter, for instance, epulis and the properly so called carcinomas, have no similarity at all. New formations, possessing all around them quite perceptible borders, do not contain any parts of normal tissue, while on the contrary, those called infiltrated, place themselves just between the elementary parts of tissue.

Of the utmost importance, however, is the distinction of benign and malign pseudoplasmas, as the name of benign is given to those which take their origin neither in cachexy, nor in any particular condition of the blood. In their very nature they must be regarded as local diseases, the effects of which only evince themselves by displacement of tissue or organs, desolation by pressure, and sometimes by pain effected therefrom. It may also happen that they result in a general disease, but then it goes on under the influence of disturbed functions, or by an extensive or protracted suppuration of a pseudoplasma. On the other hand, all these affections which originate in a general condition of specific dycrasy, or which follow, sooner or later, any such tendency, belong to the class of malign ones. Now, even here they sometimes assume the shape of local diseases, but then the effects will be seen in a peculiar composition of the blood, in the same way as hectic or pyæmic fever arises from protracted suppuration, or typhoid fever from progressive combustion of some part of the body. This fact seeks its explanation in exchange of matter during the act of nutrition of pseudoplasmas and its entering into larger or smaller blood or lymphatic vessels where the mixture of blood with elements of malign formations may already have been effected.

But with all that, it cannot be denied that benign and malign pseudoplasmas are by no means in every case strictly discernible; to judge, therefore, the degree of malignity, and to bear in mind by what symptoms the last named are mostly to be recognized during life time, we have to hold on as many points of support as the experience of practice allows to do, and although we cannot trust on the validity of signs for all formations of this kind, yet as more of the qualities hereafter described are found, the better we may conclude on their malignity.

Pseudoplasmas of this character, are usually painful or such will be the result after a certain time of existence ; for the most part, the pain exacerbates at night, so much so as to be almost intolerable, and may be spontaneous, or arise from slight pressure, or even touch. The severity of the pain depends on circumstances, as the bordering normal tissue, more or less provided with nerves, is sometimes adduced, and, therefore, pulled by the pseudoplasma ; or the development of any such new formation going on at a place where the gathering of its elements is prevented by mechanical impediments. Benign pseudoplasmas very rarely give rise to pain, and if ever this occurs, it does not last continually, because they are insensible to pressure except when they originate from a nerve, or lay near one like the neuromas and cavernous tumors.

Arising in the cellular tissue beneath the skin, or having progressed so far, malign pseudoplasmas grow together with the superficial cover and its elasticity loses at some places ; the same refers also to the serous and slimy coats.

Its form of infiltration is another proof of the malignity, as no benign pseudoplasma makes its appearance in this way, and for the most part formations of this kind grow faster than those of the last named ; but as frequently exceptions may occur, this symptom cannot be relied upon without coincidence with some others.

Furthermore, a certain point of development being attained, malign pseudoplasmas begin to soften, and burst either at a single point, or at many ones simultaneously, whether the tumor is still small or already voluminous. This, far from being always the result of inflammation, is frequently effected without any of her products, as the process itself adheres to the very nature of the tumor, the time of emolition depending on the place where the affection is located.

As well on the surface as beneath, the tissue thus breaks in, while in benign new formations it is very rarely observed on enchondroms, fibroids and lipomas, although something like happens, in many instances, by inflammation and suppuration of parts, which of course could not be confounded with it.

Glands, laying along the course of lymphatic vessels, are in no small degree found to swell, in which case their tumefaction is a purely sympathetic one, or else degeneration of their tissue has in its own way progressed. In the former, the parts, nevertheless, do not evince any more firmness and sensibility as soon as the original pseudoplasma has been destroyed, while in the latter, the part is remarkably consistent and painful. It cannot be passed by without notice, that in the first case, the glands are only infiltrated with the fluid blastema of the new formation, carried there by the lymphatic vessels, but not effecting any new organization. If ever this is the true condition, then there is every chance for metamorphosis of the germ fluid after the main evil has been extirpated. On the contrary, whenever local degeneration has already taken place, the process, in spite of all operating actions, goes on independently, whereby the glands become transformed into the very same pseudoplasma, if not something worse.

No rule can be established about the time of glands swelling in the manner and under circumstances aforesaid ; that it depends, at least, upon the slow or rapid growth, place of degeneration, distance of the nearest lymphatic glands, differences of tissue, emolition in the parenchym and bursting of the tumors has, in numerous cases, sufficiently been demonstrated. Allusion could be made, no doubt, to some analogy, perhaps in benign formations, but there, as has been already remarked, the glands never swell, unless they or the neighboring parts are placed in an inflammatory and suppurative condition, after the disappearance of which, by antiphlogistic treatment, the tumefaction likewise passes over.

Any malign pseudoplasma having at length chapped and been exposed to the external influences of the air, etc., the diseased process of vegetation goes on more rapid and unabated. It is worthy of our attention to notice that increasing volume sometimes is observed in a very small degree or not at all, because of the granulation going on in the same way as she was formed, effected, as there is no doubt, by the corrosiveness of the cancerous fluid. However benign formations may become wounded and also exposed to the air, no such luxurious growth has ever fallen under observation, if we except some kinds met with in the mouth, where the warm and humid fomentation, together with the friction by food, the presence of the teeth and tongue, etc. adds to the influences of the air.

In some instances, like the melanotic carcinoma and scirrhus, the only direction we get at hand, consists in some smaller knolls around the first one ; we are better aware of the malignity by their simultaneous development at several places of the body and various systems of the organism, though we have remembrance of many similar transactions as it regards the more innocent forms, such as warts, enchondroms, lipomas and fibroids. The existence of the former being suspected, however, we cannot omit that the general condition of health rarely escapes their deleterious effects, and cachexy in many cases, lies at the bottom. Another argument, unfortunately, why carcinomas multiply very often, points most convincingly to the inopportunity of time when extirpation has been performed.

For this reason, the very fact of such easy recurrence at the same or some other places, must be admitted to the other evidences of malignity. To beware of errors, it would scarcely be out of occasion to state, that luxurious growth found sometimes on the gum, whenever the original affection has not entirely been taken off, cannot strictly be classified among the carcinomas.

The blood source whence malign forms spring up, either is a single impure one or the cachexy is the effect and result of matters unapt of assimilation and carried out of the tumor to the blood and lymphatic vessels. The former refers in particular to the fungous cancer and fibrous, the latter to the flat carcinoma; both, of course, impair the health and appearance of the patient.

On those signs, taken up from daily observation, we may hold for any solid base to determine the true character of the various pseudoplasmata. Whatever else has been tried, to the same purpose, it is still more vague and inconsistent than are, we must confess, some points perhaps set forth above. In this respect at least, microscopic analyses are of far less avail than it might generally be supposed, indeed, of no more than the distinction of pseudoplasmas, made according to their chemical composition, albumen being pointed out as the prevailing compound in malign, lime in benign ones. Besides the almost impossibility of recurring to them before the operation has been made; there are pseudoplasmata of a malign nature which are found consisting entirely of lime. Yet, what in any way seemed reliable in regard to the detail of such researches, will be said at its proper place.

In accordance with the great majority of diseases, these degenerations also participate in the division of chronic and acute, the flat carcinoma growing most slowly, while, on the converse, the epithelial and medullary carcinomas, liable to infiltration, invade their proper locality in much shorter time than any others do. The same carcinomas, originally growing single and at one spot, are called primary, but become of a secondary character as soon as by reaction on the blood similar productions appear, first in the adjacent glands, afterwards in the interior organs of the body. Neither the preceding of a single production, in case of several organs being involved, nor the ultimate result into secondary forms, can be fixed as a general rule, because many primitive formations always remain in the same state.

As to the scale of their most frequent occurrence, they are found successively declining in the following organs: uterus, mamma, lips, stomach, tongue, colon, rectum, lymphatic glands behind the peritoneum, liver, bones, eyes, testes, ovarium, penis, œsophagus, bladder and salivary glands. Many parts never are attacked primitively, although frequently ex-contiguo, like the serous coats, jejunum, lungs and spleen. What might be said about the secondary ulcers, tends to a certain sympathetic relation, such as the transition of a carcinoma of the testes to the kidneys and their neighborhood, from the kidneys to the spleen, from the stomach to parts of the intestine, and so forth.

Together with observations of this kind, the distinction of independent and symptomatic carcinomas, in other words, the difference between cancerous tumors, with phenomena of local degeneration, and others arising from a diseased condition of the blood, demands for practice the fullest attention. However, indeed, we might be inclined to deny the real existence of any carcinoma in the shape of a purely local affection, under the belief that even the smallest carcinomatous knot ought to be regarded as the effect of cachexy, it is utterly wrong to take hold of such a notion, contradicted in too numerous instances by the very facts themselves. According to these facts, any carcinoma evinces the quality of a local affection, if circumstances explicitly coincide, as perceptible external cause of its development, proportionately slow growth, sound condition of the neighboring glands, or these may just have commenced to swell, although the degeneration existed a long time previously; the pseudoplasma being single, and not having yet increased in volume, good looks of the individual in regard to color and nutrition, and the age in which carcinomas usually appear, not yet attained. Still, the most careful investigation may lead to errors, particularly if the other points are lost sight of, and too much validity has been attributed to good nutrition. Experience mainly shows that, in spite of dyscrasy raging through the whole body, its size, or to call it the formation of fat, for a long while does not diminish at all, for which the fungous, not as much the fibrous, carcinoma must be accounted here. Altogether, fungous carcinoma exceedingly seldom deserts its rank among the general diseases, more frequent the fibrous, and most often the flat cancer, now and then the epithelial, bundle, and gelatinous pseudoplasma.

Under all reversed circumstances the tumor is bound to a general suffering, and, to use a figure, pale color of the skin, changing into yellowish gray, represents in reality a mirror, thus reflecting the concealed disease of the blood, and the presence of a degeneration in the internal organs.

All along, with facts hitherto elaborated, we now have come to cast a glance on the properly so-called carcinomatous cachexy, which, as has been already stated, originates either by retroaction of some local degeneration upon the blood mass, or precedes the cancerous tumor in the form of protopathic affection. Many particulars, like the succeeding of each other, of fungous and fibrous, medullary and gelatinous carcinoma, the change of primary into secondary ones, also the chemical analysis, seem to indicate one and the same crasis for all the various kinds of pseudoplasmata; on the other hand, there is quite a difference, inasmuch as epithelial cancer never gives rise to secondary degeneration, except in the neighboring glands. This, however, does not lessen the probability of the assertion just made. Cancerous cachexy of course cannot be deprived of phenomena of its own, but it happens, in a number of cases, that for the reason of their being entirely concealed cancerous cachexy is not even suspected, and does not awake suspicion before the new formation has already been multiplied, or suddenly grown again after extirpation. The symptoms too, by which the crasis may be recognized, are very few, and their quality scarcely enables us to separate them exactly from other general diseases. If the progress is slow, it may pass through many years, but if rapid, then a few months may suffice to effect the entire dissolution of the body.

Its slow march falls in particular under observation, when cachexy has sprung up from local nature of the carcinoma, and presents the symptoms nearly common to all diseases in the same way deleterious. Paleness, with yellowish gray color, decline of the body's volume, nutrition and muscle strength make appear the first steps for progressive emaciation, leaving its further traces by rapid, feeble pulsation, deficiency of appetite, severe pain in the tumor, sleeplessness, bad effects of the cancerous fluid upon the digestion, and through the simultaneous inherent development of gas upon the inhalation when epithelial cancer, for instance, opens into the mouth ; fever, thirst, changed mixture of the urine, anæmia, hydræmia and œdema. By this proceeding, the life being gradually more and more endangered, the occurrence of its being saved by natural exertions, such as gangrene, is an extremely rare one, and may rather be accounted for by the discontinuance of the bad cancerous actions upon the blood. In no other way the possibility of entire restoration may sometimes be realized.

Of course, there are various transitions from slow to acute progress, which, as often as cachexy precedes any cancerous formation, is almost regularly met with, the more if after the ablation of a carcinoma its blastema with diseased blood gets deposited in many other points than it at first occupied; also when the tumor has been irritated by incisions, and incited to new luxurious growth, and when the contagious blastema, or the already existing forming elements, were again poured into the blood. The same symptoms, though in much shorter succession, are peculiar to this kind.

With reference to the peculiarity of the blood in said diseases, it first needs to be fully aware of the patient being authentically affected with a carcinoma, while the blood subjected to chemical analysis can only be obtained by experimental bleeding, scarifying, and other surgical operations, or by spontaneous hemorrhage. The researches made in the laboratory of Dr. Heller led to the following results, first in respect to the examination by the microscope.

The blood corpuscles were of the utmost variety of sizes, sometimes below the normal one, sometimes increased as much as three times their original volume. We may say, however, that the same can be found in putrified blood too, and other diseases, whereon it proves no peculiar quality of carcinomas, and is missed in blood procured by scarification.

Still, in the largest number of cases, the cancerous elements could unmistakably be ascertained in the shape of round, oval cells, in volume like those found in pus, granulated, with one to three larger granuli imbedded therein, and not changeable by acetic acid. It was quite easy to get a very good sight of them by diluting the blood, free of fibrin, with distillated water, until it became impossible to discern under the microscope any blood corpuscles; in a narrow retort the mass afterwards was allowed to stay in the cold, when, after the lapse of twenty-four hours, the red fluid was poured off from the formed, though small, sediment, placed in another retort, again washed off, led to deposit, then the water decanted, and the sediment again examined.

Out of chemical experiments other statements were collaborated. No less in absolute than in relative proportion fibrin was found to be constantly on the increase. In 1000 parts of normal blood, taking a share of 3, it always averaged it from 4—13, sometimes even to 16.42. Albumen either was normal or below its usual portion, for which reason it cannot be asserted, that the cancerous dyscrasy consists in albuminosis, and that carcinomas develop themselves from albuminous blastema. The mass of blood corpuscles continually was very small, and vacillated between 56—104, their norm said about 127. All solid matters have been constantly far below their normal portions, corresponding with the diminution of the blood corpuscles, which sufficiently elucidates the paleness of such patients.

According to that there can be no doubt about the real fibrinous nature of carcinomatous crasis, the less as it agrees with the experience, that never such productions occur while mechanical impediments exist in the central organs of circulation, albuminosis being always connected therewith.

At this point we may still be allowed to make mention of some different diseases by which pseudoplasmata are, in no small degree, influenced, and of the process of healing, when, though rarely, effected by nature alone. Such pathological actions which have to be placed here, are: hemorrhage, especially occurring in very vascular structures, for instance, fungous hæmatodes and medullary carcinoma; their tender, soft tissue is apt therefrom to become pinched and lacerated. Hyperæmia and inflammation either arise spontaneously or by external causes, but appear the less whenever the tissue gives evidence of qualities heterogen to the organism; the inflammation, like all others, of course, declines frequently in the usual way, but it happens quite as often that it results into abscess, and entire or partial destruction of superficial or deep seated layers.

Gangrene, as has been said before, is seldom met with, but is one of the salutary proceedings, at least in some cases. The same effect must be attributed to the transformation into fat, if it takes in all parts of such tissue; quite often, however, these expectations are disappointed.

Organic elements of carcinomas, epulis, neuromas and gelatinous tumor, while sinking successively to decay, get changed into a yellow, untransparent, easily pressible mass, similar to tubercles, at one or many points of the tumor, and it depends equally upon the extension of this process, whether the affection loses or retains its bad effect. In enchondromas, fibroids particularly, not as often in the stroma of medullary carcinoma, ossification occurs, setting limits to their further development, however imperfect this kind of healing must be regarded. Allusion may be made too of cellular incrustation, mostly perceived in cavernous tumors, enchondromas and gelatinous carcinomas. In the last place, we have to refer to the salutary act of partial absorption, while the remaining parts undergo the process of becoming changed into fat or chalk, as well in benign tumors as in carcinomas, the stroma of which has then shrunk by absorption.

Traced as we have so far our subject in a general outline, first, by determining the nature of these affections, and afterwards describing the character of their origin and organization, their tissue, division, symptoms of malignity, predominant cachexy, diseases and natural process of healing, it is our task now to notice their classification for further detail. And here we would say, that for the practical point of view we already started from, a rapid glance all around the great circle in which new formations move, will, no doubt, scarcely be considered inadmissible. It is their distinction into benign and malign ones to which we go back again, and relying on all the facts within our reach, of practice and experience, they will be classified as seen below:

*Benign Pseudoplasmata.*

I. Epidermidal formations, { common warts, crusty warts, horns.

II. Epithelial formations,—excrescences in the mouth.

III. Tumors of adipose tissue, { on the skin, { berry like, even, ; in cellular tissue beneath the skin, ; on the slimy coat of the mouth, ; syphilitic condylomas, ; caruncles of the urethra. ; knotty excrescences on serous coats, ; on bones.

IV. Fibrous tumors, { scar-like, fibroids in proper sense, albuminous fibroids.

V. Fatty tumors, { simple lipoma, ; knotty lipoma, ; fatty tumors, combined with other formations.

VI. Cartilaginous tumor,—enchondroma.

VII. Bony tumor,—osteoïd.

VIII. Fungous hæmatodes, erectile tumors, { ragged, cavernous.

IX. Muscle-fibrous tumor or sarcoma.
X. Cysts, without parenchym.

The formations hitherto enumerated, entirely benign, belong more or less to homöoplasies; next in order are those which form the transition to the opposite character, recognized either by their ill-effects upon the lymphatic system and nutrition, or by severe pain and decline of organic strength. Which are as follows:

XI. Glandular gelatinous tumor.
XII. Neuroma.
XIII. Epulis.

*Malign Pseudoplasmata.*

XIV. Epithelial carcinoma, { flat, granular or alveolar, wart-like.
XV. Bundle-like carcinoma.
XVI. Gelatinous carcinoma, { with perceptible stroma, with unperceptible stroma.
XVII. Fibrous carcinoma.
XVIII. Medullary or fungous carcinoma, { common, raveled, melanotic.

In point of malignity and often occurrence, medullary carcinoma holds the first rank, succeeded by epithelial and fibrous carcinoma. Epithelial carcinoma, particularly in its granular structure, although sometimes too of the utmost deleterious nature, wherein, upon an average, surpassing both gelatinous and bundle carcinoma, is placed as above, for the reason of its non-conjunction with the other kinds, and some peculiarities in reference to structure. Parenchymatous cysts, or cystosarcomas, tumors, of which the nature, according to the differences of their tissue, evinces sometimes benign, sometimes malign phenomena, have to be spoken of separately.

Now, coming across any of these different forms of degenerations, the question presents itself, by what means are we able to fix the diagnosis.

The diagnosis of pseudoplasmata, no practitioner will feign not to notice it, belongs to the most difficult problems surgery is ever called upon to solve; it requires persevering study and immense experience. But even the most sagacious and learned will sufficiently often meet with embarrassments, in fact, to content himself with the notion of either of both opposed qualities, or with the reduction of the diagnosis on 2—3 species. It has yet to be remarked, that the pathological conditions humanity has ever been afflicted with, are far from being all included in the above system. Nature, on the contrary, shows herself to such a degree inexhaustible, that after the operation had been performed, and by means of the most minute anatomical examination and chemical analysis, we do not know how to assign the tumor. Gloomy as this truth may be, it can only incite to still more assiduous studies which, no doubt, will by-and-by clear up all darkness in this dominion..

In all cases, the diagnosis must be based upon the phenomena of the affection, the order in which they succeed each other, and the whole process of the disease; on the state, age, blood condition of the patient, occasional causes, if known, and the result of therapeutics hitherto observed. After the tumor has been taken off, our knowledge may, in other ways, become more complete, and similar cases in future of less trouble.

With reference to the manner how to appreciate these symptoms or phenomena, we will take notice of the following circumstances:

The volume of the new formations, which often lends us strong support, inasmuch as some, like atheromas, compared with lipomas, never overgrow a certain size; we need to know also, if the volume can be diminished by pressure, like all kinds of fungous hæmatodes, or not.

The form is either round or oval in tumors of the skin and neuromas, pear-like in fibrous polyps, irregular in many glandular gelatinous tumors and medullary carcinoma, etc.

The color of degeneration on the skin is red, blue or black; if beneath, blueness becomes transparent, as also in fungous hæmatodes, or the skin appears rather marbled as in medullary carcinoma of the mamma.

Consistency or resistance against pressure; either bone-hard: osteoids, ossified enchondromas and fibroids; or cartilage-hard: scirrhus and many fibroids, etc.; or soft, like rude flesh: fatty cellular tissue. The hardness is found on all points equal: usually in cartilaginous and bony tumors, or unequal: in many parenchymatous cysts, carcinomas in emolition, etc.

The elasticity, either great as in lipomas, or impossible to determine, as in very hard tumors, often varies at different points and times, for instance, in erectile tumors.

We have to see for apparent, obscure, or no fluctuation, whether at one or several points, or in the whole extent of the structure, whether the walls of a cavity full of fluid are tight or not.

The surface is even: in simple cysts, or uneven: in glandular gelatinous formations. The unevenness symmetrical, small, wart-like, as now and then in enchondromas; pointed, knobby, as in warts, many epithelial carcinomas; ragged: lipomas; with hemispheric greater eminences, seen sometimes in bundle-carcinomas; or fully irregular.

The kind of connection with the neighboring parts has equally to be examined, whether the mass is movable and easily shoved out of its place: atheroma, or only for some part, or not at all, as carcinomas grown together with the ribs or enchondromas growing out of bones. In regard to the connection with the skin, we are to ascertain, too, whether the latter runs movably over the degeneration and can be folded, or if somewhere strong adhesion with the tumor beneath has taken place.

The borders are either distinct or there is such a gradual transition of the tumor into the normal tissue, that to point out the end of the one and beginning of the other, is utterly impossible. In contrast to the infiltrated ones, movable tumors are of course always strictly marked.

As to the degree of sensibility, the tumor is or is not painful; the first, either by pressure, observed on the medullary carcinoma, or some hours after examination, which sometimes is the case with scirrhus; or the pain is of spontaneous character, intermittent and passing quickly, as in the first stadium of carcinomas, or rages only during the night or lasts continually, although for some moments only remitting, with exacerbations at night as in progressed periods of the flat and fibrous carcinoma.

The temperature is equal to that of adherent parts, or exalted as in fungous hæmatodes. In all other pseudoplasmata it accounts for the presence of congestion or inflammation.

It may happen that a tumor, from time to time, breaks asunder to empty a fluid without the tissue, therefore, being exposed to the air, as it occurs in the cystosarcoma of the mamma, or it is in a permanent way that the tissue becomes exposed to external influences, in which event, form, color, discharge, mass and kind of secretion, sensibility by touch and hardness of the wounded space have to be closely watched. It ought not to escape our attention, furthermore, if hemorrhage makes its appearance, if the luxurious growth on the surface is in proportion to the tissue dying in consequence of the said external effects, surpasses it, or is on the contrary excelled by the destruction, thus indicating the progress of substantial defect.

The growth advances slowly: atheroma, or rapidly: medullary carcinoma, equally: caruncles of the uretha, or with more vehemence after a certain period had been attained: carcinomas. After having progressed to a certain size, many tumors: warts, atheromas, ossified enchondromas, become in their growth confined, while others develop themselves unlimited.

Occasionally, hindrance of functions falls under observation, such as swallowing, by carcinoma of the œsophagus, motion by degenerated articulation, urine, relief of the bowels by carcinoma of the uterus.

The retroaction of a pseudoplasma on the lymphatic glands, blood composition and nutrition, has already been valued above. That in each case all present phenomena have in their apprehension to be taken together with the process itself, the succession, change and duration of each symptom is a matter of course.

For the great benefit of purely anatomical investigation, any tumor as soon as the operation has been made, ought to be examined, however hasty this might be done, because some minutes often suffice to perceive everything important. In this respect there is to consider: whether the tumor is enclosed by an integument of adipose tissue or adheres fully with other surrounding tissues; the integument, if delicate or firm, fibrous, shining, continuing itself into the tumor or not. Consistency as well as elasticity often differ remarkably of these observed during life time, just as the same regards the color. The mass itself may be transparent in some parts, or at the borders only, or nowhere.

In cutting through the tumor, the surface of the incision may deviate from the level by projecting granuli, small glands or irregular parts. Fluid pouring out appears in different colors and is obtained by pressure or scraping with the knife; while the tumor, provided with many or few blood vessels, is cut in, some crackling may often be heard. Of the tumor no texture is either visible or it consists of fibrous or glandular organization, is often easier found out after maceration in water during 24—48 hours, when by this proceeding, the appreciation of the time and change of putrefaction and color, leads us also to further conclusions.

To avoid errors in microscopical analysis, the tumor always should be examined in its fresh condition or after it has been preserved for the length of time in Goodby's fluid, 48 ℥ water, 4 ʒ chloride of sodium, 2 ℥ alum, 4 grs. sublimate, which is far preferable to water or alcohol. To get a better sight of delicate blood vessels, it requires more concentrated chloride of sodium, just as the use of chromic, nitric acid, tincture of iodine or chromate of potasse, will be found advantageous for condensing the transverse section of the object. In the very act of preparing the latter we may get some hints of what we are just searching about. Thus, if the whole breaks into roundish particles, it is quite an allusion to its granular structure, while in feazing it directs to real fibrils or to the stringing of other elements along the fibrous form. Border lines similar to arcs almost correspond with glandular structure, like the dark points with the concentration of granular cells.

However often it might be expected to define the diagnosis by microscopical research, it must not be overlooked, that with all subtile variety of organization there is as little use of it, as the same kind of pseudoplasma differs in tissue according to the period of development, place, and vitality adhering to its germ layer. Therefore, what is viewed by the usefulness of the microscope, tends a good deal more to the exploration of the most delicate particles of tissue, their development and reciprocal arrangement. In the same manner, attended with much difficulties, are the investigations by means of chemistry, because all matters deriving out of the blood and liable to organize into plasma are subjected to continual change, thereafter constituting parts, though in regard to structure distant from each other during lifetime, turning out to be in striking similarity.

Elsewhere, some remarks have already been dropped about the influence of nature in behalf of the issue of pseudoplasmata, which but for exceedingly rare exceptions is totally inefficient. What, on the other hand, lies in the power of surgery, enters too much into the combined detail of other matters to dwell any longer on this general introduction, and shall with no inferior purpose be the subject of the next part.

[To be continued.]

